# Simultaneous quantification of estrogens and glucocorticoids in human adipose tissue by liquid-chromatography-tandem mass spectrometry

**DOI:** 10.1016/j.jsbmb.2019.105476

**Published:** 2019-12

**Authors:** Sofia Laforest, Mélissa Pelletier, Nina Denver, Brigitte Poirier, Sébastien Nguyen, Brian R. Walker, Francine Durocher, Natalie Z.M. Homer, Caroline Diorio, André Tchernof, Ruth Andrew

**Affiliations:** a CHU de Québec-Université Laval Research Center (Endocrinology and Nephrology Division), School of Nutrition, Faculty of Agriculture and Food Sciences, Université Laval, Québec, Canada; b Quebec Heart Lung Institute, Québec, Canada; c Mass Spectrometry Core, Edinburgh Clinical Research Facility, Queen’s Medical Research Institute, Edinburgh, United Kingdom; d Institute of Cardiovascular and Medical Sciences, College of Medical, Veterinary and Life Sciences, University of Glasgow, University Avenue, Glasgow, United Kingdom; e Strathclyde Institute of Pharmacy and Biomedical Sciences, University of Strathclyde, Cathedral Street, Glasgow, United Kingdom; f CHU de Québec-Université Laval Research Center (Oncology Division), Université Laval Cancer Research Center and Department of Surgery, Faculty of Medicine, Université Laval, Québec, Canada; g Centre des maladies du sein Deschênes-Fabia, Hôpital Saint-Sacrement, Québec, Canada; h University/BHF Centre for Cardiovascular Science, Queen’s Medical Research Institute, University of Edinburgh, 47, Little France Crescent, Edinburgh, EH16 4TJ, UK; i Newcastle University, Newcastle upon Tyne, NE1 7RU, UK; j CHU de Québec-Université Laval Research Center (Endocrinology and Nephrology Division), Université Laval Cancer Research Center and Department of Molecular Medicine, Faculty of Medicine, Université Laval, Québec, Canada; k CHU de Québec-Université Laval Research Center (Oncology Division), Université Laval Cancer Research Center and Department of Social and Preventive Medicine, Faculty of Medicine, Université Laval, Québec, Canada

**Keywords:** ^13^C_3_-E1, 2,3,4-[^13^C_3_]-estrone, ^13^C_3_-E2, 2,3,4-[^13^C_3_]-17β-estradiol, DCM, Dichloromethane, E, Cortisone, E1, Estrone, E2, Estradiol, ESI, Electrospray ionization, Et_2_O, Diethyl ether, EtOAc, Ethyl acetate, EtOH, Ethanol, D_4_-F, 9,11,12,12-[^2^H_4_]-cortisol, FA, Formic acid, GC–MS/MS, Gas chromatography-tandem mass spectrometry, HPLC, High-performance liquid chromatography, IS, Internal standards, LC–MS/MS, Liquid chromatography-tandem mass spectrometry, LLE, Liquid-liquid extraction, LOQ, Limit of quantitation, LOD, Limit of detection, MeOH, Methanol, MPPZ, 1-(2,4-dinitro-phenyl)-4,4-dimethylpiperazinium, MRM, Multiple reaction monitoring, MTBE, Methyl *t*-butyl ether, OFN, Oxygen-free nitrogen, PPZ, 1-(2,4-dinitro-5-fluorophenyl)-4-methylpiperazine, RME, Relative mean error, RSD, Relative standard deviation, SNR, Signal-to-noise ratio, SPE, Solid-phase extraction, UHPLC, Ultra-high-performance liquid chromatography, Estradiol, Estrone, Cortisol, Cortisone, Derivatization, Adipose

## Abstract

The presence of estrogens, androgens and glucocorticoids as well as their receptors and steroid converting enzymes in adipose tissue has been established. Their contribution to diseases such as obesity, diabetes and hormone-dependent cancers is an active area of research. Our objective was to develop a LC–MS/MS method to quantify bioactive estrogens and glucocorticoids simultaneously in human adipose tissue. Estrogens and glucocorticoids were extracted from adipose tissue samples using solid-phase extraction. Estrogens were derivatized using 1-(2,4-dinitro-5-fluorophenyl)-4-methylpiperazine (PPZ) and methyl iodide to generate a permanently charged molecule (MPPZ). Steroids were separated and quantified by LC–MS/MS. The limit of quantitation for the steroids was between 15 and 100 pg per sample. Accuracy and precision were acceptable (<20%). Using this method, estradiol, estrone, cortisone and cortisol were quantified in adipose tissue from women with and without breast cancer. This novel assay of estrogens and glucocorticoids by LC–MS/MS coupled with derivatization allowed simultaneous quantification of a panel of steroids in human adipose tissue across the endogenous range of concentrations encountered in health and disease.

## Highlights

• Steroid analysis using LC/ESI-MS/MS is reliable and sensitive.
• ESI efficiency of estrogens is limited.
• Chemical derivatization of steroids improves their detectability.
• Simultaneous quantification of E2, E1, cortisol and cortisone was achieved.

## 1 Introduction

**Fig. 1:**
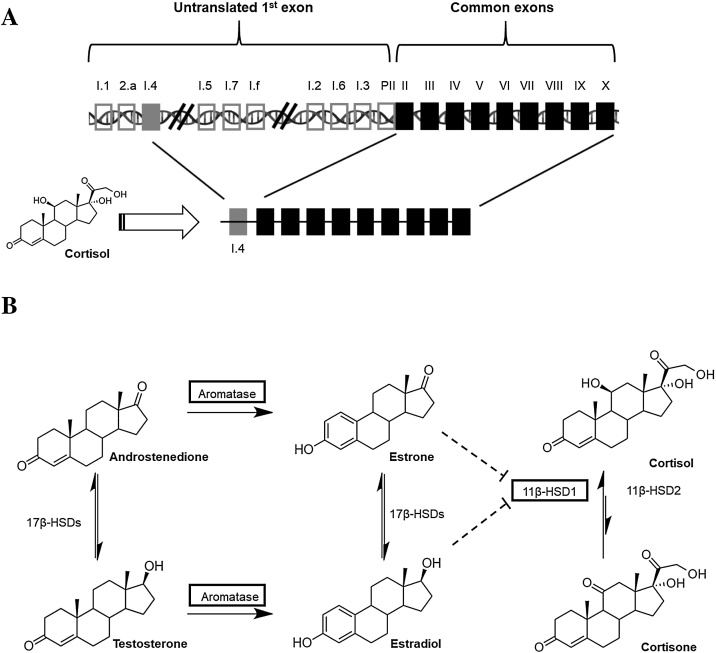
Pathways of glucocorticoid and estrogen metabolism and hypothesized cross-regulation in adipose tissue. A) Alternative splicing of the rate-limiting enzyme aromatase (CYP19A1). Tissue-specific aromatase expression in normal adipose tissue is conferred by promoter I.4 which possesses a glucocorticoid response element. B) Androstenedione and testosterone are converted into estrogens by the action of aromatase. Androstenedione and testosterone as well as estrone and estradiol are interconverted by the action of several 17β-HSDs. Cortisone is converted into active cortisol by the action of 11β-HSD type 1 (reductase) which predominates over 11β-HSD type 2 in adipose tissue. Higher concentrations of estrogens may inhibit the activity of 11β-HSD type 1. Expression of enzymes in black squares are increased in the adipose tissue as a function of adiposity.

Adipose tissue is an active endocrine organ and a site of storage for steroids due to their lipophilicity. We and others have described several steroid-converting enzymes localized in adipose tissue [1] and proposed their importance in modulating adipose tissue function e.g. adipocyte hypertrophy and lipid storage. Glucocorticoid and estrogen concentrations and their respective activation enzymes, 11β-hydroxysteroid dehydrogenase 1 (11β-HSD1) and aromatase are both increased in adipose tissue in obesity, although little is known about their interactions and cross-regulation [2]. Increases in estrogen concentrations in breast adipose in obesity may be of importance for local tumor growth [1]. Glucocorticoids can increase androgen-to-estrogen conversion in adipose tissue through activating the glucocorticoid response element on exon I.4 of the aromatase gene, a well-established mechanism [3] (Fig. 1). Accordingly, aromatase and 11β-HSD1 expression in subcutaneous adipose tissue are positively associated [4]. However, evidence from rodent studies suggests that high estrogen concentrations inhibit the expression of 11β-HSD1 [[5], [6], [7], [8]]. These apparently conflicting results warrant further study of adipose tissue steroid homeostasis by measurement of the active steroids rather than inferring function from transcript levels of the enzymes.

Accurate quantification of steroid hormones in adipose tissue is difficult. Mass spectrometry is the gold standard analytical approach [9], but adipose tissue presents significant challenges as a matrix. High concentrations of lipidic compounds sharing similar physico-chemical properties to those of steroids can cause substantial ion suppression and interfere with the steroid signal. Removing interfering compounds may help increase signal to noise of the peaks of the steroids of interest, but one must also consider the concomitant signal loss that may occur during processing. Of particular note, the use of different sample preparation and analytical approaches for specific steroid hormones makes it difficult to allow the direct comparison among studies [2,[10], [11], [12], [13]].

In the context of research, curation of large biobanks of human adipose samples is difficult and collection of sufficient clinical material (e.g. more than 1 g) per patient in various disease states is challenging, especially in fat depots of interest (visceral, breast). Immunoassays, such as radioimmunoassays and enzyme-linked immunosorbent assays, have the advantage of high sensitivity and so do not use large amounts of samples, but they can be limited by specificity [9,14]. Liquid chromatography-tandem mass spectrometry (LC–MS/MS) is an attractive alternative as the analytical technique for development of an extraction and quantification protocol for steroids in adipose tissue, as it is already the gold standard for analysis of steroid panels in plasma [15,16]. It typically offers shorter run times per sample compared to gas chromatography-tandem mass spectrometry (GC–MS/MS). Assays based on LC–MS/MS had previously been used for quantifying glucocorticoids in adipose tissue in our laboratory [17], but were not optimized for estrogens.

Our aim was to develop a new LC–MS/MS method to detect and quantify estrogens (17β-estradiol (E2) and estrone (E1)) as well as glucocorticoids (cortisol, cortisone) in adipose tissue. Considering the limited amount of tissue available, derivatization of estrogens was deemed necessary as E2 and E1 are present in 10-100-fold lower concentrations than glucocorticoids, in plasma and adipose tissue [2,[10], [11], [12], [13],[15], [16], [17]], and have a poor ionization profile. We adapted a validated, highly efficient derivatization approach for estrogens in serum developed by Nishio et al. [18] for use in adipose, drawing from modifications we had made to the method to quantify a wider panel of estrogen in plasma [19].

## 2 Materials and methods

### 2.1 Standards and solvents

E1, E2, and 17α-estradiol (17α-E2) were obtained from Steraloids, Inc (Newport, USA). Cortisone, cortisol, iodomethane (≥99%) and internal standards (IS), 2,3,4-[^13^C_3_]-17β-estradiol and 2,3,4-[^13^C_3_]-estrone (^13^C_3_-E2, ^13^C_3_-E1 respectively) were from Sigma-Aldrich, Inc. (St. Louis, USA). 9,11,12,12-[^2^H_4_]-cortisol (D_4_-F) was from Cambridge Isotopes Laboratory (England, UK). 1-(2,4-dinitro-5-fluorophenyl)-4-methylpiperazine (PPZ) was from TCI chemicals (Chuo-ku, Tokyo, Japan). HPLC grade glass distilled solvents (methyl *t*-butyl ether, MTBE; acetone; ethyl acetate (EtOAc); water) were from Fisher Scientific UK Limited (Leicestershire, UK). AR grade ethanol (EtOH) and HPLC grade glass distilled solvents (acetonitrile; acetic acid; diethyl ether (Et_2_O); dichloromethane (DCM); hexane; methanol (MeOH)) and LCMS grade solvents and chemicals (acetonitrile; formic acid (FA); water) were from VWR (England, UK).

### 2.2 Adipose tissue samples

Adipose tissue samples for method development and validation originated from breast adipose tissue obtained from women undergoing reduction mammoplasty. Aliquots (∼200 mg) were stored at −80 °C. The study protocol was approved by the Research Ethics Committees of Laval University Medical Center (DR-002-136). All patients signed a written, informed consent prior to surgery.

### 2.3 Standard solutions

Glucocorticoids, estrogens and IS (1 mg) were dissolved in MeOH (1 mL) and stored at -80 °C. Working solutions (0.0001–1000 pg/mL) were prepared by serial dilution on the day of use.

### 2.4 Extraction method

All glass tubes and vials (borosilicate glass tubes, Fisherbrand; glass tubes, Corning; glass vials, Scientific Laboratory Supplies) containing adipose tissue were preconditioned with the corresponding solution required at this step (1 mL) and MeOH (1 mL) followed by vortexing (1 min) and drying (15 min, 60 °C). Adipose tissue (∼200 mg) enriched with IS (5 ng of ^13^C_3_-E2, ^13^C_3_-E1 and D_4_-F) were homogenized (Model Pro 200, ProScientific, Inc, Monroe, CT, USA) in EtOH:EtOAc (1 mL; 1:1) and immediately frozen on dry ice and stored at −80 °C overnight. Blank and standard solutions were prepared concomitantly in EtOH:EtOAc. The following morning, samples were thawed on wet ice and sonicated (8 × 15 s bursts with 1-minute gaps; Ultrasonic cleaner, Branson Ultrasonic Inc, Danbury, CT, USA). Samples were subjected to centrifugation (3200 *g*, 45 min, 4 °C; Heraeus Megafuge 16R, ThermoFisher Scientific, Germany). The supernatant was transferred into a new glass tube and reduced to dryness under oxygen-free nitrogen (OFN, 60 °C). Samples were resuspended in aqueous MeOH (30% v/v, 5 mL). Solid-phase extraction was carried out after conditioning the C18 Sep-Pak columns (12cc, 2 g; Waters, Wilmslow, UK; MeOH (2 × 10 mL), followed by water (2 × 10 mL)). The adipose extract was loaded, and the column washed with water (10 mL) followed by aqueous MeOH (5%, 10 mL). Steroids were eluted using MeOH:CH_3_CN (1:1, 10 mL) into clean glass vials. The eluate was dried under OFN before derivatization of the estrogens.

### 2.5 Generation of MPPZ derivatives

**Fig. 2:**
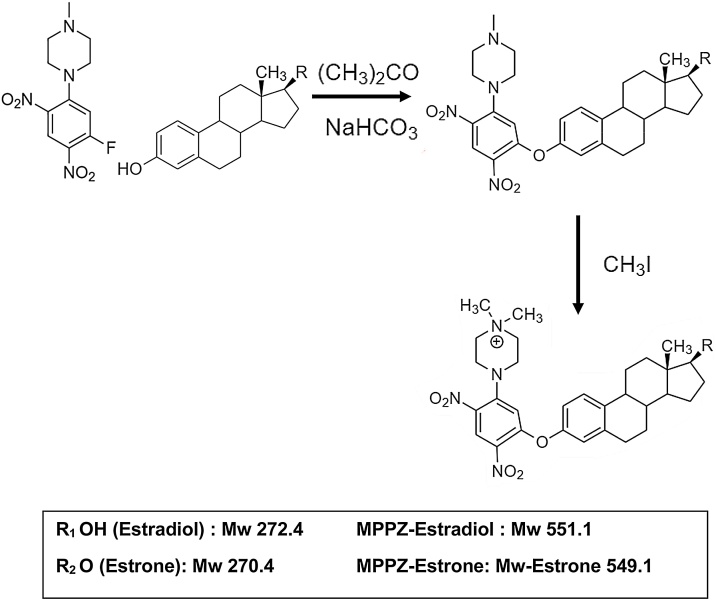
Formation of estrogen derivatives.

MPPZ derivatives were prepared as previously reported [19]. Briefly, acetone (70 μL), sodium bicarbonate (10 μL, 1 M), and PPZ (10 μL in acetone, 1 mg/mL) were added to the standard/extracted sample and incubated (1 h, 60 °C). The sample was reduced to dryness under OFN, followed by addition of iodomethane (100 μL) to the residue (2 h, 40 °C) [19]. After reduction to dryness under OFN, samples were then dissolved in LC–MS grade water:acetonitrile (70 μL; 70:30). A schematic representation of the generation of MPPZ derivatives of E1 and E2 is shown in Fig. 2 [18,19].

### 2.6 Instrumentation

Cortisone, cortisol, E1 and E2 were quantified by LC–MS/MS, using a UHPLC Shimadzu Nexera 2 system (Kyoto, Japan) coupled to a Sciex QTRAP® 6500+ (SCIEX, Warrington, UK) equipped with a Turbospray interface and operated with Analyst software v1.6.3. MS conditions are described in Table 1, with ion spray voltage (5500 V) and source temperature (500 °C) and GS1 (414 kPa) and GS2 (276 kPa). The compound-dependent parameters are also described in Table 1. Optimal MS/MS precursor-product transitions and voltages were used, assigned following direct infusion of individual solutions, as previously described [19,20].

**Table 1:** Mass spectrometric conditions for analysis of analytes and internal standards by positive ion electrospray ionization.

	Molecular Weight g/mol	MRM transition for monitoring		Declustering potential (V)	Collision energy (V)	Cell exit potential (V)
		Precursor ion (*m/z*)	Product ion (*m/z*)			
**Analytes**						
Cortisone	360.5	361.1	163.1	81	31	26
Cortisol	362.5	363.1	121.2	76	31	8
Estrone-MPPZ	549.6	549.1	502.3	100	59	20
Estradiol-MPPZ	551.7	551.1	504.3	100	129	8
**Internal standards**						
D_4_-Cortisol	366.5	367.3	121.0	166	41	54
^13^C_3_-Estrone-MPPZ	552.6	552.3	505.3	100	39	15
^13^C_3_-Estradiol-MPPZ	554.6	554.3	507.3	100	35	15

Key: MPPZ, 1-(2,4-dinitro-phenyl)-4,4-dimethylpiperazinium; MRM, Multiple reaction monitoring; V, Volts.

### 2.7 Chromatographic conditions

Standards of glucocorticoids and MPPZ estrogens were injected individually to confirm chromatographic resolution using an ACE 2 Excel C18-PFP (150 × 2.1 mm, 2 μm, ACT Technologies, Aberdeen, UK) column.

At a constant flow rate of 0.5 mL/min, the chromatography conditions began with 90:10 water with 0.1% FA (solution A) and acetonitrile with 0.1% FA (solution B) which was maintained for 1 min. This was followed by an 11-min linear gradient to 50% B, which was maintained for 2 min, before returning to 10% B by 15 min, again maintaining for 3 min to re-equilibrate. The column and auto-sampler temperatures were 40 °C and 15 °C, respectively. Injection volume was 30 μL.

### 2.8 Assay validation

#### 2.8.1 Apparent extraction efficiency

Different compositions, volumes and types of elution solvent were tested, namely, DCM, MeOH and MeOH:acetonitrile (1:1). Recoveries of steroids from adipose tissue and standard solutions were assessed by comparison of signal intensities between samples pre- and post-spiked with IS (5 ng; before homogenization and after solid-phase extraction respectively).

#### 2.8.2 Assessment of matrix effects

Ion suppression was assessed by comparing signal intensity of IS post-spiked into extracted adipose tissue samples with that of aqueous steroid solutions following derivatization. To reduce ion suppression without compromising recovery, washes with MeOH (0–30%) were assessed. A hexane wash was also tested.

#### 2.8.3 Specificity

Extracted ion chromatograms were carefully examined according to the retention times of IS for interferences by other endogenous compounds in adipose tissue extracts, which could introduce inaccuracies in quantitation.

#### 2.8.4 Linearity

Blank samples and aliquots containing estrogens (5, 7.5, 10, 15, 25, 50, 100, 200, 500, 1000 pg/sample), glucocorticoids (50, 75, 100, 150, 250, 500, 1000, 2000, 5000, 10000 pg/sample) and combined IS (5 ng) were analyzed by LC–MS/MS. Calibration curves were plotted as the peak area ratio (standard/IS) versus amount of analytes (glucocorticoids or estrogens). Calibration lines of best fit were acceptable if the regression coefficient, r, was >0.99. Weightings of 1, 1/x and 1/x^2^ were compared and 1/x weighting selected to reduce errors at low amounts of analyte.

#### 2.8.5 Accuracy and precision

The precision and accuracy were assessed using standard solutions prepared on the same and different days. The precision was calculated as the Relative Standard Deviation (RSD) (standard deviation/mean × 100), and % accuracy was the Relative Mean Error (RME) ((mean measured value − theoretical value)/theoretical value × 100); precision was accepted with RSDs 20% and RME 100 ± 20% [21].

#### 2.8.6 Limit of detection and quantitation

The signal-to-noise ratio (SNR) was calculated from peak areas of steroids and adjacent background noise (over the same time window as the peak width). The limits of detection were assigned at a SNR ≥ 3 [21].

Replicate aliquots (7.5, 15, 25, 50, 1000 pg/sample and 0.075, 0.15, 0.25, 0.5, 10 ng/sample) of estrogens and glucocorticoids, respectively with IS were prepared as above and analyzed. The LOQ was calculated as the amount affording precision and accuracy of ∼20% or less [21].

### 2.9 Method application

The presence of glucocorticoids and estrogens was assessed, and their amounts quantified in breast adipose tissue from healthy women (n = 6) and breast cancer patients (n = 17) using the validated method.

## 3 Results and discussion

Analysis of steroids in small biopsy samples of adipose tissue from clinical studies is desirable. Here we report a method allowing both glucocorticoids and estrogens to be assessed in single adipose tissue samples, applied here to breast tissue in the setting of cancer. Challenges existed in combining these steroids in one assay due to different dynamic ranges in concentration, as well as different chemical properties between phenolic and non-aromatic steroids. The use of MPPZ derivatization enabled detection of estrogens, without compromising quantitation of glucocorticoids.

### 3.1 Extraction

Both liquid-liquid extraction (LLE) followed by solid-phase extraction (SPE) or SPE on its own have been used to recover estrogens and glucocorticoids from adipose tissue samples in previous publications [2,[10], [11], [12],^22^]. SPE was our favoured approach here, as extraction column technologies have been developed to reduce ion suppression particularly with complex matrices. Although analyte specific, LLE presents drawbacks with reports of high variability across experimenters due to manual errors [23].

Alternative conditions were tested to improve recovery of the main analytes of interest i.e. cortisone, cortisol, E1 and E2. Homogenization solutions of either EtOAc, EtOH:EtOAc (1:1), Et_2_O:EtOAc (2:1) or water were tested to solubilize the steroids. When water was used, this was then followed by LLE comparing three different organic solvent solutions used in previous publications with estrogen extraction protocols: Et_2_O:EtOAc (2:1) [24], Et_2_O [25] or MTBE [26]. Under these circumstances, poor recovery rates were achieved; <15% for ^13^C_3_-E2 and <50% for ^13^C_3_-E1. Recovery for D_4_-F from the homogenate into EtOAc was highest (60–70%) as previously reported [17,20]. Addition of EtOH with EtOAc lowered the recovery for cortisol (D_4_-F) only slightly (around 5%), but increased recovery of ^13^C_3_-E2 and ^13^C_3_-E1 significantly. The best recovery rate for both estrogens was achieved using EtOH:EtOAc (1:1) (>60%).

Following homogenization, shattering the tissue by dripping it through acetic acid, and steps involving sonication and centrifugation were also assessed to enhance extraction efficiency [17,20]. Sonication and longer centrifugation time improved recovery by 5–10%, but “acetic acid dripping” of tissue led to a loss of the estrogens [17,20]. Final sample clean-up by SPE was assessed comparing reversed phase matrices with polymeric sorbent (Oasis HLB®). As previously reported, reversed-phase C18 (BondElut® (2 g, 12cc) and Sep-Pak® (2 g, 12cc) C18 columns) showed better recovery rate and lower matrix effect for steroids isolated from adipose when compared to polymeric sorbents, unlike from plasma [19,26,27]. In our hands, recovery was not different across the two reversed-phase C18 columns tested, although sample preparation was quicker with Sep-Pak® compared to BondElut®, due to a faster flow.

To decrease ion suppression by cleaning the sample further, a variety of washing steps with Sep-Pak® columns were tested, aiming to maintain recovery. Washes tested included, water (2 × 10 mL); water (1 × 10 mL) followed by aqueous 5%, 10%, 20% or 30% MeOH (1 × 10 mL); water (1 × 10 mL) followed by aqueous 5% MeOH (1 × 10 mL) and hexane (1 × 10 mL); and finally, water (1 × 10 mL) followed by hexane (1 × 10 mL). Use of aqueous MeOH washes, in the range 10% to 30%, before elution (as performed when recovering estrogens from plasma using Oasis MCX cartridges [19]) led to significant loss of analytes with the Sep-Pak® columns. A 5% MeOH wash did not affect recovery but improved signal (by diminution of ion suppression) significantly. Washing with the more lipophilic solvent, hexane, led to a loss of analytes.

Concomitantly, elution solutions (MeOH; CH_3_CN; MeOH:CH_3_CN (1:1); DCM) and volumes (5–10 mL) were tested. DCM is recommended in elution using supported liquid extraction of estrogens [28], but it did not completely elute estrogens from the reversed phase C18 columns. The same was true for acetonitrile used alone. MeOH is the manufacturer’s choice of elution solvent for Sep-Pak® columns, however a mixed phase of MeOH:CH_3_CN (1:1) led to reduced matrix effect and improved recovery for estrogens compared to MeOH alone. MeOH alone yielded better recovery of cortisol, as previously published [17,20], and using MeOH:CH_3_CN (1:1) led to a further loss of ∼5% but this was deemed acceptable for the combined assay, given that glucocorticoids were more abundant. Of note, measurements of recovery were increased when collection tubes were preconditioned with the elution solvent (*K. Soma, personal communication, 2018*).

### 3.2 Chromatographic conditions

**Fig. 3:**
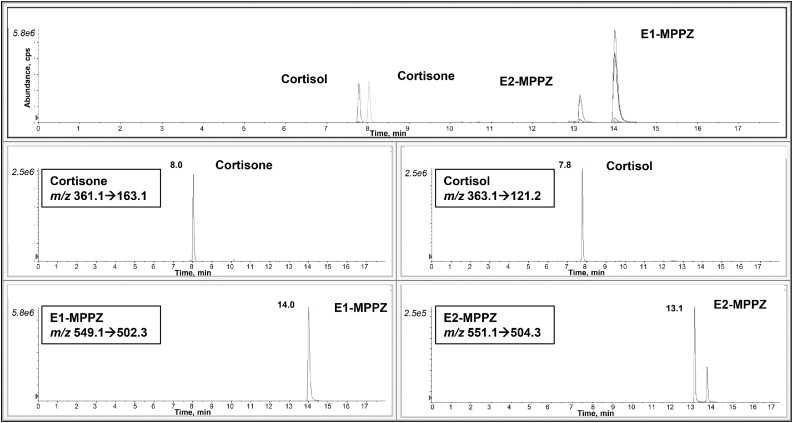
Mass chromatograms of glucocorticoids and MPPZ derivatives of estrogens following analysis of an unextracted solution of standards, 1000 pg/sample. Total Ion Chromatograms and the corresponding extracted ion chromatograms showing resolution of cortisone, cortisol and derivatives of estrone and estradiol, by retention time and mass transition.

Chromatographic conditions were based on those developed by Denver and collaborators [19] for the analysis of estrogens in plasma. Using the same gradient and column, we could not separate cortisol and cortisone, which was necessary as cortisol may suffer isobaric interference from natural isotopologues of cortisone. Following changes to the gradient, the two glucocorticoids were separated, without affecting the separation of derivatized estrone and estradiol (Fig. 3). The gradient was achieved more rapidly and maintained for a shorter period than Denver et al. [19], who also analyzed estrogen metabolites. Increasing the column temperature to 40 °C increased the resolution of the glucocorticoids. Of note, initially we observed a shift in the retention time, tracked by the isotopically labelled IS, between extracts of standards and those of adipose tissue, but this drift was eliminated by addition of high organic washes (95% CH_3_CN) after four-five adipose tissue samples and was most likely to be due to build-up of lipid residues in the column. However, we cannot rule out that this is also due to build-up of the derivatizing agents as we were faced with similar issues with plasma samples [19]. To further improve robustness, centrifugation (3000 *g*, 5 min) of the derivatized sample prior to injection was also introduced. Under these circumstances, the retention time was maintained between 0.24 and 1.18% (1.2–30 s) during a batch size of 30 with 23 adipose samples. It would be valuable to assess robustness after larger numbers of biological samples, but batch sizes of 40 are currently the maximum achievable per single run.

### 3.3 Specificity

**Fig. 4:**
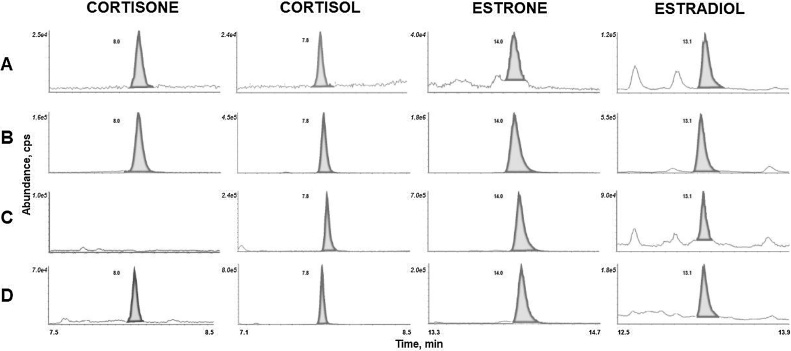
Mass chromatograms of glucocorticoids and MPPZ derivatives of estrogens extracted from adipose tissue. Extracted ion chromatograms at (A) the lower and (B) upper limit of quantitation and in adipose tissue (C) from control women and (D) women with breast cancer.

Baseline chromatographic separation of both glucocorticoids and derivatives of estrogens was achieved using aqueous standards (Fig. 3). Stable isotope-labelled E2, E1 and cortisol were selected from previous applications [19,20]. Isotopically labelled IS can both introduce and suffer from isobaric interferences, but this was pre-empted in the design of the chromatographic method. We also confirmed that inactive 17α-E2 does not elute at the same time as active 17β-E2. The use of three stable isotope standards allowed for confidence in identification in the biological matrix. When applied to adipose tissue samples, the chromatographic regions close to the retention time of the analytes were free from any interferences which may disrupt peak shape (Fig. 4). Of note, some interferences higher than SNR = 3 were observed before adding high organic washes between adipose samples. Peaks were symmetrical for all analytes and IS without any indication of closely eluting compounds. Qualifier transitions of *m/z* 363.1→91.1 (cortisol), *m/z* 361.1→77.0 (cortisone), *m/z* 551.1→58.1 (E2) and *m/z* 549.1→72.0 (E1) may be added should further reassurance of specificity be required.

### 3.4 Linearity

**Fig. 5:**
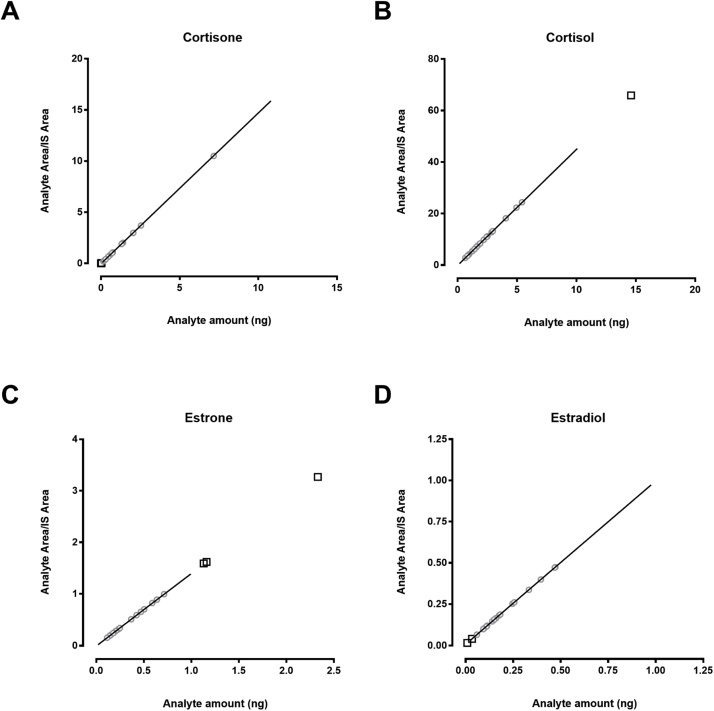
Calibration curves of glucocorticoids and MPPZ derivatives of estrogens following extraction. A) Cortisone, B) Cortisol, C) Estrone and D) Estradiol. Regression lines (representing the range covered by the standard curve) were fitted with a 1/x weighting. Grey circles represent values of patient samples falling in the linear range and black squares represent those values requiring extrapolation.

Linear standard curves of cortisone, cortisol, E1-MPPZ and E2-MPPZ were generated (Fig. 5). A mean r-value > 0.99 was achieved for analytes with a weighting of 1/x (Table 2). The linear ranges were similar to those used in other methods quantifying those steroids in human adipose tissue, albeit not in combination [2,13].

**Table 2:** Limits of Detection, Quantitation and Linearity of Response.

Metabolite	IS	Recovery of IS (%)	LOD (pg/sample)	LLOQ (pg/sample)	ULOQ (pg/sample)	R	RT RSD (%) Endogenous (IS)	RT Delta (s)
E1	^13^C_3_-E1	82	10	15	1000	0.99	1.18 (1.18)	30
E2	^13^C_3_-E2	62	10	25	1000	0.99	1.03 (0.99)	24
Cortisone	D_4_-F	NT	50	75	10 000	0.99	0.24	1.2
Cortisol	D_4_-F	47	17	100	10 000	0.99	0.25 (0.26)	1.2

Key: E1, Estrone; E2, Estradiol; IS, Internal standard; LOD, Limit of detection; LLOQ, Lower limit of quantitation; ULOQ, Upper level of quantitation; NT, Not tested; RSD, Relative standard deviation; RT, Retention time.

### 3.5 Accuracy and precision

The values for intra-assay precision and accuracy (Table 3) were acceptable (<20% RSD for precision and <±20% accuracy) at low and high points of the calibration curve. Cortisone showed less precision and accuracy than cortisol, most likely attributable to the use of cortisol IS (D_4_-F) for cortisone and not labelled cortisone IS. D_8_-cortisone is available commercially and could be introduced in the future. The precision and accuracy of the upper cortisone points could be improved by use of an unweighted standard curve.

**Table 3:** Accuracy and precision of the method.

Metabolite	Target (pg/sample)	Mean (pg/sample)	Precision (RSD %)	Accuracy (RME %)
E1	15	17	9.9	10.0
	25	28	13.2	12.11
	50	49	11.9	2.8
	1000	1018	5.2	1.8
E2	25	29	12.8	17.4
	50	47	11.2	2.8
	1000	1075	3.4	5.8
Cortisol	*75	67	6.0	11.1
	^#^150	152	6.5	1.6
	250	244	12.3	2.1
	500	460	13.8	8.0
	10,000	8962	9.0	10.3
Cortisone	*75	61	11.3	19.2
	^#^250	213	12.0	6.1
	500	415	17.2	17.0
	*10,000	8143	20.8	18.6

Key: E1, Estrone; E2, Estradiol; RSD %, Relative standard deviation (standard deviation/mean × 100); RME %, Relative Mean Error ((mean measured value - theoretical value)/theoretical value × 100); n = 6 replicates unless otherwise specified: ^#^ n = 5 replicates; *n = 4 replicates.

### 3.6 Limits of detection and quantitation

The LODs for the four analytes of interest are shown in Table 2. We report an LOQ of 15 pg and 25 pg on column for E1-MPPZ and E2-MPPZ, respectively (Table 2). Adjusting for a generic mass of 200 mg of adipose tissue, this equates to ∼275 pmol/kg and ∼459 pmol/kg. This is a higher LOQ for E2 than the ones reported in negative ESI [11,12] and in GC–MS/MS [13]. However, those other methods are not directly comparable as they did not combine estrogen and glucocorticoid extraction and thus could focus the instrumental conditions to a greater degree. Due to the permanently charged moiety of the derivative produced, we used ESI in positive mode which has inherently more noise than negative mode [11,12]. Positive ESI was necessary for the combined approach as glucocorticoids would not readily ionize in negative mode. Care was taken to ensure that cortisone and cortisol were unaffected by the derivatization process as expected, because the nucleophilic substitution with PPZ in the presence of a base requires an activated phenolic hydroxyl group. Aliphatic hydroxyl groups in E2, cortisone and cortisol do not react with analogues of Sanger’s reagent such as PPZ [18]. In screening experiments, we did not see change in amount of D_4_-F measured in derivatized vs underivatized adipose extracts or aqueous standard solutions or any detriment to its SNR. As reported by Hennig et al., the use of only one extraction column may also explain the lower sensitivity of our combined method [13].

Despite slightly higher LOQs, our method achieved higher recovery, especially for E2, as well as reduced matrix effects, leading to quantifiable E2 in breast adipose tissue, even in postmenopausal women. Further reductions in ion suppression were difficult to achieve because upon assessment of elution fractions (1 mL), we found that components causing ion suppression occurred primarily in the same fraction that contained the estrogens. In summary, it is unlikely that adding more steps during sample preparation by SPE would remove those interferences, improve the signal and lower the LOQ, as they appear to possess very similar characteristics to E1 and E2. However, other approaches such as supported liquid extraction may afford new opportunities [29].

Linear range and LOQ of cortisol and cortisone extracted from adipose tissue are not commonly reported in publications [2]. This may be due to the ease with which these more abundant steroids can be detected in adipose tissue, but this information is valuable to compare methodologies. Methlie et al. reported a LOQ of 200 pmol/kg and a range from 200 pmol/kg to 200 nmol/kg [30]. Our LOQ values for cortisone and cortisol, 75 pg and 100 pg, represent ∼1040 and ∼1380 pmol/kg which are ∼5-fold of those reported values. However, our values fall into their interquartile range and largely in the upper range of the calibration curve. Interestingly, they performed LLE instead of SPE and reported a recovery higher than 95%, although they pre-spiked after homogenization of the tissue, compared to other glucocorticoids-only extraction methods with recoveries of ∼70% [17,20] in which the pre-spiking occurred before homogenization.

### 3.7 Method application

The method was applied to samples from healthy women undergoing reduction mastectomy and breast cancer patients undergoing partial mastectomy. We were able to detect and quantify estrogens in more than 90% of our samples using around 200 mg of adipose tissue. Cortisol was detected in all breast adipose tissue samples and cortisone in most. Of note, cortisone was undetected in 5 samples, 4 of which were from women without breast cancer, although the number of samples is too small to draw firm conclusions and not the purpose of this report. A few samples generated data higher than the ULOQ, suggesting that validation of a higher point would be advisable moving forward. Data points higher than the ULOQ were observed from breast adipose tissue from both control women and cancer patients.

Calculated amounts of cortisone and cortisol as well as estrone were in the same range as previously reported in subcutaneous and visceral adipose tissue [2] or in breast adipose tissue [2,13]. E2 levels were higher than expected by 10-fold, but this is in comparison with a very limited number of studies available in breast adipose tissue [2,13]. Interestingly, when E2 levels in breast adipose tissue are reported, levels often fall below LOQ and LOD, which was not the case with our assay. For example, Hennig and collaborators reported a LOD of 50 pg/g for all estrogens but reported a median adipose tissue concentration of 40 pg/g for E2 [13].

## 4 Conclusion

In summary, concomitant detection and quantification of cortisone, cortisol, E1 and E2 was achieved in breast adipose tissue. The combined analysis of derivatized and underivatized steroids was possible due to the specificity of the PPZ for the phenolic group of the estrogens and allowed for quantification of those steroids with low ionization potential in positive ESI in a single biopsy. This profile could most likely be extended by addition of estrogen metabolites such as the 4-hydroxyestrogens as well as underivatized androgens. This novel approach will allow quantification of estrogens and glucocorticoids in breast adipose tissue to elucidate the complex relationship of those steroids in the breast cancer paradigm.

## Funding statement

The study was supported by operating funds from the CMDO to AT. SL is the recipient of PhD scholarships from *Fonds de recherche du Québec-santé* (FRQS) and the Canadian Institutes of Health Research (CIHR). SL’s internship in RA’s lab was made possible thanks to the support of the Institute of Nutrition and Functional Foods and the scholarship from the International internship program of the *Fonds de recherche Nature et Technologies du Québec* (FRQNT) as well as a Michael Smith Foreign Study Supplement travel scholarship. RA is funded by the Wellcome Trust. BRW is a Wellcome Trust Senior Investigator. CD holds an Investigator Awards (Senior) from the FRQS. ND is funded by a BBSRC iCASE award (BB/N503691/1).

## Declaration of Competing Interest

AT is the recipient of research grant support from Johnson & Johnson Medical Companies and Medtronic for studies unrelated to this publication.
